# Event and Comment

**Published:** 1912-09-15

**Authors:** 


// DENTAL REGISTER.

Vol. LXVI. September 15, 1912. No. 9

### EVENT AND COMMENT.

INDEPENDENT DENTAL JOURNALISM. In these days of agitation for uninfluenced motives in politics and religion, it seems that medical and dental literature has caught the infection. A fierce war of editorial writing is waging in the medical journals between the American Medical Association interests and the sectarian journals. Some caustic and interesting statements are being printed, that seriously reflect on the dignity and purity of an honored profession. For many years there has been an agitation in the dental profession for a journal, that will print the professions current literature and that is not owned by a dental supply house. Up to now none has had a long life. There is a strong prospect that the National Dental Association will this year take steps to reorganize its methods of conduct so as to increase its income with the idea of having sufficient funds to enable it to print its own proceedings in its own journal. We sincerely trust that the plan contemplated may be adopted and that we may have a journal issued by the national body that is free from all possible selfish interests. We are curious enough to see it even though it may be the downfall of our own interests. It certainly ought to do a wonderful lot of good for the profession, and as we have tried to spend our life for the professions best interests, as we have conscientiously seen our way, we shall gladly give way to any organization that promises better and purer things, professionally. We are willing to be a part of the sacrifice if need be. In truth however, we do not anticipate an early demise on this account. There is to-day a large place for all the dental journals we have; not one of them however, is doing the work it should do, but largely because of the fact that the profession is not inclined to literary endeavors. If a new journal or many of them can create a desire for good professional literature and create also a demand for it, this will react on the existing journals to make thém better. Let's have a national journal by all means, we can well afford it, and it can not possibly harm any of our existing institutions of value or usefulness.

THE AMERICAN DENTAL JOURNAL. This journal has taken on a new existence under the able management and editorial control of that indefatigable worker Dr. B. J. Cigrand of Chicago. The *American Journal* has had a varied existence, being published under almost every known form of ownership and editorship. It is again owned, edited and published as an independent journal. It is true that it carries advertising otherwise it could not exist, but its editor seems to have a notion that he can use the trade influence for professional ends and he appears to be doing so. From the editorial work it is not apparent that he is catering to his advertisers. He demonstrates his ability to write interesting editorials and to secure valuable contributions from the best professional men without embarrassment. With his strong personality, long experience as a writer and professional worker we shall anticipate great things and abundant success for the *American Journal*. We certainly wish its editor success and congratulate the profession and the cause of dental literature that Dr. Cigrand has entered the field of dental journalism.

